# Hypoxia-Induced Neuroinflammation in Alzheimer’s Disease: Potential Neuroprotective Effects of *Centella asiatica*

**DOI:** 10.3389/fphys.2021.712317

**Published:** 2021-10-14

**Authors:** Aqilah Hambali, Jaya Kumar, Nur Fariesha Md Hashim, Sandra Maniam, Muhammad Zulfadli Mehat, Manraj Singh Cheema, Muzaimi Mustapha, Mohd Ilham Adenan, Johnson Stanslas, Hafizah Abdul Hamid

**Affiliations:** ^1^Department of Human Anatomy, Faculty of Medicine and Health Sciences, Universiti Putra Malaysia, Serdang, Malaysia; ^2^Department of Physiology, Faculty of Medicine, Universiti Kebangsaan Malaysia, Cheras, Malaysia; ^3^Department of Biomedical Sciences, Faculty of Medicine and Health Sciences, Universiti Putra Malaysia, Serdang, Malaysia; ^4^Department of Neurosciences, School of Medical Sciences, Universiti Sains Malaysia, Kubang Kerian, Malaysia; ^5^Universiti Teknologi MARA, Jengka, Malaysia; ^6^Department of Medicine, Faculty of Medicine and Health Sciences, Universiti Putra Malaysia, Serdang, Malaysia

**Keywords:** neurodegenerative disease, Alzheimer’s disease, hypoxia, neuroinflammation, *Centella asiatica*, neuroprotective, NF-κβ, Nrf2

## Abstract

Alzheimer’s disease (AD) is a neurodegenerative disorder that is characterised by the presence of extracellular beta-amyloid fibrillary plaques and intraneuronal neurofibrillary tau tangles in the brain. Recurring failures of drug candidates targeting these pathways have prompted research in AD multifactorial pathogenesis, including the role of neuroinflammation. Triggered by various factors, such as hypoxia, neuroinflammation is strongly linked to AD susceptibility and/or progression to dementia. Chronic hypoxia induces neuroinflammation by activating microglia, the resident immune cells in the brain, along with an increased in reactive oxygen species and pro-inflammatory cytokines, features that are common to many degenerative central nervous system (CNS) disorders. Hence, interests are emerging on therapeutic agents and plant derivatives for AD that target the hypoxia-neuroinflammation pathway. *Centella asiatica* is one of the natural products reported to show neuroprotective effects in various models of CNS diseases. Here, we review the complex hypoxia-induced neuroinflammation in the pathogenesis of AD and the potential application of *Centella asiatica* as a therapeutic agent in AD or dementia.

## Introduction

Millions around the world are suffering from various forms of neurodegenerative diseases (Kim et al., 2013; Zahra et al., 2019). Neurodegenerative disease is classified as a deteriorating medical condition, which leads to progressive degeneration and death of neurons in the peripheral or central nervous system (CNS), that clinically manifest as irreversible cognitive deterioration and/or movement disturbances (Kim et al., 2013; Dugger and Dickson, 2017). Neurodegenerative diseases are incurable, as the majority of the neurons are not replaced when they are damaged, although neurogenesis was reported in some parts of the brain (Ming and Song, 2012). According to the World Health Organization (WHO), dementia is a syndrome with the presence of multiple cognitive malfunctions including memory loss, inability to process thoughts and difficulties in judgement and language deficits (Kempler and Goral, 2008; Prince et al., 2014; WHO, 2020). Dementia, one of the prominent symptoms of many neurodegenerative diseases, is predominantly caused by Alzheimer’s disease (AD; Cai et al., 2014; Prince et al., 2014; Lall et al., 2019).

AD may result from a combination of aetiologies, but the most widely accepted pathological hallmarks are deposition of amyloid plaques and neurofibrillary tangles (Hardy and Allsop, 1991; Goedert, 1993; Lane et al., 2018). Other than these two, neuroinflammation has also been implicated in the pathogenesis of AD (Morales et al., 2014). Based on the neuropathological and neuroradiological studies of this disease, evidence suggests that neuroinflammatory responses are mounted in the presence of external insults or injuries to the neurons. Hypoxia is one of the main causes of neuroinflammation (Lall et al., 2019). Other factors include enhanced Aβ production, increased phosphorylation of tau, enhanced production of reactive oxygen species (ROS) and abnormal mitochondrial function (Zhang et al., 2019).

Despite being a significant global health concern, to date, only five drugs – four are cholinesterase inhibitors and one is NMDA blocker – for symptomatic management of AD have been approved (Briggs et al., 2016). However, none alters the course of the disease. As AD is known to have a multifactorial pathogenesis, there are various potential targets that can be targeted in developing the therapies rather than focusing on its classic amyloid-tau pathological hallmarks. In the past decades, a plethora of studies has extended their research interest in the potential application of natural products as a therapeutic agent for AD. In doing so, many plant extracts or natural bioactive compounds were reported to have memory-enhancing effects (Chiroma et al., 2019; Mairuae et al., 2019; Ma et al., 2020; Prom-In et al., 2020). Among these, *Centella asiatica* (CA), a medicinal plant with known anti-oxidant and anti-inflammatory properties, serves as a potential lead in identifying natural products with neuroprotective effects.

## Pathophysiology of Alzheimer’s Disease

AD mainly affects the elderly (over 60years old); however, there are some rare cases (less than 0.5%) of familial AD (usually early-onset) affecting individuals between the age of 30 to 50years old (Bateman et al., 2011; Ranjan et al., 2018). Although the frequency of familial AD is exceptionally low, it still provides some crucial pathogenetic avenues of AD for researchers to further explore its complexity. The familial AD may occur in individuals who had multiple mutations in three genes – amyloid precursor protein (APP), presenilin 1 (PSEN1) and presenilin 2 (PSEN2; Lane et al., 2018). Meanwhile, the sporadic form of AD (typically late-onset) is a result of the complex interaction between genetic, lifestyle and environmental factors. Clinical features of AD are attributed to pathological changes commonly found in the hippocampus, entorhinal cortex and neocortex areas of the brain (Praticò et al., 2000). Two major hypotheses were linked to the pathogenesis of AD, the amyloid-cascade hypothesis (ACH; Hardy and Allsop, 1991) and the tau hypothesis (Goedert, 1993; Lall et al., 2019).

The ACH hypothesis has been widely accepted for over two decades. Based on this hypothesis, abnormal processing of APP due to mutations in gamma and beta-secretase enzymes results in an imbalance between amyloid-beta (Aβ) production and clearance, which leads to aggregation and accumulation of Aβ peptides (Hardy and Allsop, 1991; Querfurth and LaFerla, 2010). Then, Aβ peptides trigger a cascade resulting in some fundamental pathological features of AD: amyloid senile plaques (Glenner and Wong, 1984; Hardy and Allsop, 1991) and neurofibrillary tangles (NFTs) of hyperphosphorylated tau protein (Grundke-Iqbal et al., 1986; Goedert, 1993), along with microglial activation, cerebral amyloid angiopathy, dystrophic neurites, neutrophil threads and astrogliosis (Briggs et al., 2016; Lane et al., 2018). The ACH hypothesis indicates that Aβ accumulation and deposition are upstream of tau phosphorylation in AD (Briggs et al., 2016; Lane et al., 2018).

In a normal physiological condition, tau is a microtubule-linked protein, which regulates the stability of tubulin assemblies and is abundantly found in neurons of the CNS (Kametani and Hasegawa, 2018; Lall et al., 2019). According to the tau hypothesis, hyperphosphorylation of tau leads to its alteration by appearing as twisted fibrils with ∼80nm periodicity into paired helical filaments and NFTs (Crowther and Wischik, 1985), resulting in neuronal death and dementia (Zempel and Mandelkow, 2014). Mutations in tau gene have been linked to some dementing disorders, such as frontotemporal dementia and parkinsonism linked to chromosome 17 (FTDP-17; Spillantini et al., 1997), but its link to AD is yet to be elucidated (Kametani and Hasegawa, 2018). Therefore, at present, the ACH is a more well-established initial trigger of AD compared to the tau hypothesis.

Other than these two major hypotheses of AD, there are other mechanisms which can contribute to the underlying pathophysiology of AD, such as cholinergic hypothesis, neuronal excitotoxicity, neuroinflammation and more. In AD patients’ brains, cholinergic deficits due to deficits of enzyme acetylcholinesterase (AChE) have been reported (Talesa, 2001). Acetylcholine plays a crucial role in memory and learning; thus, the cholinergic hypothesis of AD was formed (Briggs et al., 2016). Though depletion of cholinergic innervation is a late feature of the neurodegenerative cascade, it is worth curbing the symptoms of AD to improve life quality of the patients. Therefore, acetylcholinesterase inhibitor (AChEI) has been tested at clinical levels to treat AD. AChEI inhibits the cholinesterase enzyme, which hydrolyse acetylcholine into choline and acetic acid at the synaptic cleft, potentiating cholinergic transmission (Colovic et al., 2013). Currently, there are few AChEIs that have been used to delay the AD progression and symptoms, such as donepezil, rivastigmine and galantamine (Colovic et al., 2013; Briggs et al., 2016). A recent meta-analysis study involving 14,705 participants has demonstrated that galantamine 32mg, galantamine 24mg, donepezil 5mg and donepezil 10mg were more effective than other interventions in improving the cognitive functions of AD patients, with the surface under the cumulative ranking curve (SUCRA) values of 93.2, 75.5, 73.3 and 65.6%, respectively (Zhang et al., 2020).

Furthermore, neuronal excitotoxicity, caused by either overstimulation of N-methyl-D-aspartate (NMDA) receptor or overexposure to the glutamate neurotransmitter, is responsible for the progressive loss of neuronal cells in AD (Choi, 1992; Lipton, 2005). The loss of cholinergic neurons is influenced by this toxicity due to excessive calcium influx into the cells leads to cognitive decline in AD (Muir et al., 1993; Ferreira-Vieira et al., 2016). In addition, inflammatory response in the CNS, also known as neuroinflammation, which induced by Aβ accumulation, which later exacerbated by the presence of tau proteins. Glial cells, such as astrocytes and microglia, regulate the neuroinflammatory response in the CNS. Microglial cells are the resident macrophages of the CNS; they represent around 10% of the CNS population and play a crucial role in neurogenesis, neuronal plasticity and regeneration. Microglial cells also act as the first line of defence in brain injury (Calsolaro and Edison, 2016). Aggregate and soluble Aβ species overload, together with hyperphosphorylated tau proteins, affect neuronal function and initiate the inflammatory response of microglia in initiating the AD pathology (Bolós et al., 2017). A pro-inflammatory process that is mainly regulated by activated microglia could precede AD pathology (Calsolaro and Edison, 2016). Several pathological changes have been reported in the AD brain, including decreased cerebral blood flow (CBF), changes in white matter and excessive iron (Chen et al., 2010). The decrease in cerebral blood flow results in hypoperfusion, causing cerebral hypoxia, which is a typical vascular component among the AD risk factors (Hassan and Chen, 2021). Prolonged and severe hypoxia leads to memory impairment and neuronal loss. Patients with stroke have greater risk of developing AD. Up to 1/3 of post-stroke survivors suffer from dementia (Mijajlovic et al., 2017), which is most commonly caused by AD, vascular dementia and mixed dementia (Hassan and Chen, 2021).

## Neuroinflammation

Previous notion of immune-privileged CNS was partly due to the presence of blood–brain barrier (BBB), lack of lymphatic drainage and immune-incompetent microglia (Barker and Billingham, 1978; McGeer and McGeer, 1995; Carson et al., 2006). However, in the early 1900s, it was disproved when the inflammatory response was reported in brain injuries (Bell et al., 1994; McGeer and McGeer, 1998; Latta et al., 2015). At present, it would be more precise to acknowledge that CNS is privileged with specialised immunity, including microglia and astrocytes (Ransohoff et al., 2003; Latta et al., 2015; Russo and McGavern, 2015). CNS is immune-competent and actively interacts with the peripheral immune system (Carson et al., 2006).

The term ‘neuroinflammation’ illustrates the brain injury that triggers an inflammatory response, followed by activation and accumulation of glial cells (microglia/astrocytes). Neuroinflammation is the secondary response to infections or trauma or even micro-level insults. This secondary response starts with a short-lived and acute neuroinflammatory response in the CNS in response to noxious stimuli, which appears to be protective at a controlled rate. Over time, when the noxious stimuli persist, a chronic neuroinflammatory response occurs, causing cumulative damage to the CNS (Streit et al., 2004). The duration of an inflammatory response, be it short-term (days to weeks) or long-term (months to years), can determine whether it is beneficial or harmful to the brain (Morales et al., 2014; Abudukelimu et al., 2018). A prolonged secondary response is thought to cause more neuronal loss over time compared to the initial injury (Akiyama et al., 2000; Morales et al., 2014).

Several pro-inflammatory and anti-inflammatory cytokines have been identified in the regulation of neuroinflammatory responses. Pro-inflammatory cytokines are a group of proteins that promote inflammatory responses include, but not limited to, interleukin 1 beta (IL-1β), interleukin 6 (IL-6), tumour necrosis factor-alpha (TNF-α), interleukin 20 (IL-20), interleukin 33 (IL-33), leukaemia inhibitory factor (LIF), interferon-gamma (IFN-γ), granulocyte-macrophage colony-stimulating factor (GM-CSF), monocyte chemoattractant protein 1 (MCP-1), interleukin 11 (IL-11), interleukin 12 (IL-12), interleukin 17 (IL-17), interleukin 18 (IL-18) and interleukin 8 (IL-8; Akiyama et al., 2000; Cai et al., 2014). Meanwhile, anti-inflammatory cytokines are a group of proteins that inhibit the inflammatory responses, such as interleukin 4 (IL-4), interleukin 10 (IL-10) and interleukin 13 (IL-13; Akiyama et al., 2000; Cai et al., 2014). Some proteins may act as both pro- and anti-inflammatory cytokines, such as transforming growth factor-beta 1 (TGF-β1; Akiyama et al., 2000; Cai et al., 2014; Morales et al., 2014). Neuroinflammation is generally associated with high levels of pro-inflammatory cytokines, which has been correlated with neurotoxicity and progression of neurodegenerative diseases (Frank-Cannon et al., 2009; Morales et al., 2014). In AD, elevated expression of inflammatory mediators includes cytokines, chemokines, prostaglandin E2, nitric oxide (NO), ROS, nuclear factor kappa beta (NF-κβ), macrophage colony-stimulating factor (M-CSF) and MCP-1 were demonstrated around Aβ peptide deposits and neurofibrillary tangles (Akiyama et al., 2000; Morales et al., 2014), suggesting a complex interconnection between neuroinflammation and neurodegeneration.

The activated microglia and reactive astrocytes promote Aβ deposition (Delaby et al., 2015). Several studies in mouse models have shown that amyloid deposition was increased in inflammatory conditions (Guo et al., 2002). In one study, the induction of systemic inflammation in transgenic mice with amyloidogenic mouse Saa1 protein expression was found to enhance the amyloid deposition along with an increase in pro-inflammatory cytokines in the brain. Moreover, cytokines upregulated β-secretase mRNA protein and enzymatic activity (BACE1; Sastre et al., 2003), a key enzyme in neuronal Aβ formation (Sastre et al., 2006). Transcription of β-secretase also seems to be increased by TNF-α-activated NF-κβ signal, resulting in increased Aβ production (Chen et al., 2012). On the other hand, Lim et al. (2013) reported that Aβ fragments causes inflammatory response in the brain that leads to production of multiple pro-inflammatory mediators (Akiyama et al., 2000; Lim et al., 2013; LaRocca et al., 2021). Also, the expression of multiple receptors of pro-inflammatory mediators, including cytokines, chemokines and damage signals, on astrocytes was upregulated within this inflammatory response (Krasowska-Zoladek et al., 2007), indicating that a secondary inflammatory response occurs as astrocytes are being activated in response to those pro-inflammatory mediators (Morales et al., 2014). Hence, both Aβ deposition and neuroinflammation can be the initiator to one and another causes these detrimental conditions may worsen in the long run, which contribute to a cascade of other pathological hallmarks of AD, and eventually develop AD.

### Microglia in Neuroinflammation

Microglia are continuously motile even in an ‘inactivated’ state and often play a key role in immune surveillance by closely monitoring the microenvironment of CNS. Two-photon imaging studies illustrated that microglia utilises their processes to rapidly and autonomously patrol the CNS microenvironment (Davalos et al., 2005; Nimmerjahn et al., 2005). Owing to their dynamic profiles, microglia interact with other neuronal elements to monitor brain activity and regulate the brain networks (Zhao et al., 2018). Microglia show macrophages-like behaviours in the CNS, as they produce cytokines and chemokines as a first line of inflammatory response. In the presence of noxious stimuli, microglia initiate the inflammatory responses and restructure themselves into a condensed ‘ameboid’ form (Lynch, 2009). In this form, they become phagocytic and express toll-like receptors that signify the initiation of inflammatory response. Once the microglia are ‘activated’, they promptly change their transcriptional states to produce inflammatory cytokines and chemokines, which initiate the recruitment of peripheral leukocytes to the CNS (Zhou et al., 2006). Besides, ATP is also responsible as a signalling molecule that intervenes the interactions among numerous brain cells. These extracellular ATP, which activates purinergic (P2Y) receptors, appear to be a chemoattractant as they are responsible for a rapid microglial response at the injury sites (Davalos et al., 2005). Hence, activated microglia rearrange their cytoskeletal to accommodate P2Y receptor expression pattern on cell surface, which allow them to migrate to injury sites (Russo and McGavern, 2015) and enhance their phagocytic efficiency (Davalos et al., 2005; DiSabato et al., 2016).

When an acute inflammatory injury occurs in the brain, there is an initial defensive response from the glial cells to repair the tissue damage. However, if the ‘stimulus’ persists, a chronic inflammatory condition develops, thus contributing to neuronal dysfunction, injury, and loss (Streit et al., 2004). Post-mortem analysis of the brains of AD patients showed reactive microglia colocalised with amyloid plaques. Several amyloid peptides, fibrils and APP are potent glial activators that trigger an inflammatory response and microglial release of neurotoxic cytokines (Calsolaro and Edison, 2016). Upon binding to microglial cell surface receptors, the Aβ peptides stimulate the NF-κβ-dependent pathway and activate extracellular signal-regulated kinase and mitogen-activated protein kinase (MAPK) pathways, hence triggering expression of pro-inflammatory genes, ROS and chemokines, therefore contributing to neuronal/synaptic injury (Ho et al., 2005). Both soluble and aggregate forms of Aβ seem to induce NADPH oxidase-mediated priming in microglial cells, resulting in a release of ROS that contributes to neurotoxicity (Schilling and Eder, 2011; Calsolaro and Edison, 2016).

Activation of microglia can be classified into two, the M1 and M2 phenotypes. The M1 phenotype is pro-inflammatory and predominantly expressed at the neuroinflammation sites. In contrast, the M2 phenotype is immunosuppressive, which includes both alternative activation and acquired deactivation states (Tang and Le, 2016). The alternative activation primarily reacts to either IL-13 or IL-4, which then promotes resolution of inflammation and tissue repair. Meanwhile, acquired deactivation caused by either apoptotic cell uptake or anti-inflammatory cytokines exposure leads to alleviation of acute inflammation (Orihuela et al., 2016; Tang and Le, 2016; Zhang et al., 2017). Under pathological conditions, M1 microglia has been proposed to dominate at the injury sites, while M2 microglia may appear later as it is associated with tissue repair processes with anti-inflammatory profile, such as IL-10 and TGF-β, and extracellular matrix molecules (Michell-Robinson et al., 2015; Tang and Le, 2016; Bolós et al., 2017). M2 microglia employs the coordinated regulation of anti-inflammatory mediators and dampens the M1 pro-inflammatory responses, which consequently results in immunosuppression and neuroprotective effects. Although both phenotypes have different roles, microglia shift from one phenotype to another in maintaining neuroinflammatory state in normal physiological conditions, but are prone to be in M1 state under pathological conditions, such as AD.

In the hippocampus of PS1M146L/APP751SL transgenic AD mice, the existence of an age-dependent microglial phenotypic change has been shown, from M2 activation state with Aβ phagocytic capabilities (at 6months) to M1 phenotype (expressing TNF-α and related factors) at 18months of age. This switch coincided with excessive levels of soluble Aβ oligomers that produced deleterious effects on neuronal cells (Jimenez et al., 2008; Tang and Le, 2016). In addition, M2 microglia with YM-1-labelled which surrounded the plaques displayed the Aβ phagocytic capabilities (Jimenez et al., 2008). YM-1 is a marker of the alternative differentiation in peripheral macrophages, which indicates the M2 activation of microglia (Edwards et al., 2006; Bolós et al., 2017). Upon attenuation of the phagocytotic process by pro-inflammatory cytokines including IFN-γ, IL-1β and TNF-α, the activated microglia are most likely switched from M2 into M1 activation state (Koenigsknecht-Talboo and Landreth, 2005). However, in most cases, microglia in AD patients exhibited heterogeneous activation phenotypes. In fact, cortical tissue from the Tg2576 transgenic mouse for amyloid deposition model and AD patients showed a hybrid profile of alternative activation (M2) and classical activation (M1) genes. The authors found that M2 activation genes, including arginase I (AGI), mannose receptor (MRC1) and chitinase 3-like 3 (YM1), and M1 activation genes, such as TNF-α and nitric oxide synthase 2 (NOS2), co-existed in the cortical tissues of both transgenic mouse models and AD patients (Colton et al., 2006; Bolós et al., 2017).

## Hypoxia

Older people with systolic hypertension, severe head injury with loss of consciousness and increased exposure to tobacco smoking are at a higher risk to develop a hypoxic brain, either directly or induced by neuronal ischemia. Neuronal ischemia is due to disruption of neurovascular coupling (Khan and Davies, 2008), resulting in vascular oxidative stress and inflammation, which in turn reduce the cerebrovascular function resulting in cerebrovascular diseases (Iadecola, 2013). The risk factors for cerebrovascular diseases, including hypertension, insulin resistance, diabetes, obesity, hyperhomocysteinemia and hyperlipidaemia, are strongly correlated with vascular dementia rather than AD (Chui et al., 2012). However, the hypoxia caused by vascular insufficiency can facilitate Aβ production by activating the APP cleavage enzyme β-secretase activity (Sun et al., 2006). Indeed, both cerebral ischemia and reduced cerebral perfusion are seen in early AD or patients at risk of AD with a resultant increase in amyloid deposition in neocortical areas (Ly et al., 2012; Gao et al., 2013; Iadecola, 2013). Hence, hypoxia is believed to be one of the crucial predisposing factors in the development of AD, through neuroinflammation as well as vascular oxidative stress.

Hypoxia is a condition when there is deprivation of oxygen supply to maintain normal physiological function in cells (Manninen and Unger, 2016). Cells respond to hypoxia by inducing levels of hypoxia-inducible factors (HIFs), an important transcription factor that is known as a master regulator of low oxygen tension and glucose metabolism (Ashok et al., 2017; Hassan and Chen, 2021). Hypoxia can be classified into mild, moderate and severe, depending on its duration, such as acute, intermittent and chronic. Acute hypoxia may occur at a short period up until 48h due to a transient reduced blood flow followed by a critical reduction of normal oxygen level. Chronic hypoxia results from a long period of oxygen deprivation which may last for a few days or even weeks (Terraneo and Samaja, 2017; Hernandez-Gerez et al., 2019). Meanwhile, intermittent hypoxia happens when there is a repetitive oxygen desaturation-reoxygenation cycle and becomes chronic when the cycles last for weeks (Sapin et al., 2015).

Similar to inflammation, hypoxia can be beneficial or detrimental, depending on its severity and duration. Both acute and intermittent hypoxia are neuroprotective (Lall et al., 2019), whereas chronic hypoxia affects cellular metabolism, ATP production, Ca^2+^ homeostasis and generation of ROS and inflammation (Chen et al., 2018). Hypoxic condition is initiated by a range of cardiovascular problems, respiratory diseases, haematological diseases, respiratory dysfunction, medications and/or environmental conditions, that result in extended episodes of continuous or intermittent chronic hypoxia (Peers et al., 2009; Zhang et al., 2019). Additionally, the severity of the hypoxic condition depends on the existence of pre-existing diseases, including stroke or acute cerebral ischemia that causes the acute hypoxic condition, whereas chronic respiratory disease and sleep-disordered breathing resulted in chronic hypoxia (Zhang et al., 2018). Chronic hypoxia, especially chronic intermittent hypoxia with repeated exposures to low oxygen and reoxygenation, may have deleterious effects on AD pathogenesis, which increases the levels of oxidative stress and inflammation (Kim et al., 2013). However, acute hypoxia exerts its protective effects in the cardiovascular and nervous system with the involvement of HIF (Zhang et al., 2019).

There are two HIF subunits, the inducible HIF-α and a constitutive HIF-β. Levels of HIF-α in cells are post-transcriptionally regulated by four different types of oxygen-sensitive hydroxylases; three of them are prolyl-hydroxylases domain proteins (PHD1, PHD2 and PHD3) and one factor-inhibiting HIF (FIH) called asparaginyl hydroxylase (Chen et al., 2018). HIF-2α shares 48% amino acid homology with HIF-1α and binds to similar promoter sites but differs in the cofactors it recruits. In normoxic conditions, PHDs are active and catalyse the proteasomal degradation of HIF-1α subunits. Meanwhile, FIH hydroxylates asparaginyl residues in HIF-1α and HIF-2α, thus blocking protein interactions between the HIF-transactivation domains and p300/CBP, a coactivator of target gene transcription of the HIF complex (Palazon et al., 2014). Once oxygen level is dropped (hypoxic condition), the PHDs and FIH are inactivated, hence causing HIF-1α accumulation and binding to HIF-1β, forming a heterodimeric complex of HIF molecule (Palazon et al., 2014; Hassan and Chen, 2021). This transcriptional complex binds to hypoxia-responsive elements (HREs), within the promoter regions of target genes, and transactivates gene expression that regulates the adaptive response to hypoxia (Gaspar and Velloso, 2018). HIF-1α is ubiquitously expressed in most cells, whereas HIF-2α is more localised to endothelial cells. In line with this, HIF-1α has been reported to play a key role in the initial response to hypoxia, whereas HIF-2α regulates the hypoxic response during chronic hypoxic exposure. A prolonged accumulation of HIF-2α indicates the occurrence of adaptation to chronic hypoxia (Patel and Simon, 2008; Hassan and Chen, 2021).

Neurons communicate with each other *via* synapses. Acute hypoxia decreases synaptic activity in various parts of the brain, whereas chronic hypoxia causes neuronal cell loss and death. The neuronal and synaptic viability are essentially controlled by several signalling pathways that involving calcium ions (Ca^2+^) and chloride ions (Cl^−^) as messengers (Léveillé et al., 2008). Under chronic hypoxia condition, the neuronal excitability and membrane depolarisation can be increased, which leads to abnormal release of neurotransmitters including glutamate (Silverstein et al., 1986; Dallas et al., 2007). The disruption of glutamate reuptake during chronic hypoxic exposure leads to the high extracellular glutamate concentrations, which then facilitates the influx of Ca^2+^ and CI^−^ into neurons (Silverstein et al., 1986). As a result, a cascade of pathological events occurs, including synaptic and mitochondrial dysfunction, protease and lipase activation and osmotic swelling, and eventually neuronal death (Rothman and Olney, 1987; Qaid et al., 2021). Because of the high density of glutamate receptors on its pyramidal neurons, the CA1 region of the hippocampus is thought to be particularly prone to chronic intermittent hypoxia (CIH) damage (Maiti et al., 2008). Furthermore, the CIH reduces the levels of phosphorylated form of cAMP response element-binding protein (CREB) transcription factor (Goldbart et al., 2003), that leads to a lowering of CREB transcriptional targets, such as brain-derived neurotrophic factor (BDNF), resulting in cognitive dysfunction (Hattiangady et al., 2005). In line with this, previous studies reported that exposure to hypoxia triggers memory deficits through involvement of various mechanisms (Muthuraju et al., 2010, 2011). Particularly, imbalance in both pro- and anti-oxidative enzymes (Jayalakshmi et al., 2007), neuronal apoptosis in the hippocampus, cortex and striatum (Maiti et al., 2008), abnormal in glutamate neurotransmission (Hota et al., 2008) and alteration in cholinergic system (Muthuraju et al., 2009) play a crucial role in impairment of spatial working memory during hypoxia exposure (Qaid et al., 2020).

### Hypoxia and AD

In pathogenesis of AD, hypoxia enhances a shift in amyloid-β precursor protein (APP) processing towards the amyloidogenic pathway by downregulating the function of α-secretase but upregulating the function of β-secretase and γ-secretase (Muche et al., 2015; Salminen et al., 2017). The α-, β- and γ-secretase are important proteinases that cleave APP into Aβ peptides. In normoxic conditions, α-secretase cleaves the APP at the Aβ domain and inhibits the production of toxic Aβ peptides. Instead, in hypoxic conditions, HIF-1α stimulates the transcription of β-secretase 1 (BACE1) gene *via* the hypoxia-response element of the BACE1 promoter and interacts with γ-secretase complex resulting in elevation of their activities. As a result, the amyloidogenic processing of APP is augmented, thus producing toxic Aβ peptides excessively (Salminen et al., 2017). During hypoxia, both hippocampus and cerebral cortex are prone to the formation of cerebral amyloid angiopathy which correlates with Aβ peptide toxicity; reflects the pathological progress of AD and could be a consequence of increased neuronal and endothelial Aβ generation (Salminen et al., 2017).

Hypoxia also inhibits the expression and activity of an amyloid-degrading peptidase, neprilysin (NEP) in rats’ cortical neurons, which increases the accumulation of Aβ peptides (Fisk et al., 2007). NEP is a membrane-bound zinc-dependent metalloendopeptidase, which is commonly located in presynaptic terminals of, particularly, hippocampal, and neocortical neurons (Zhang et al., 2017). The NEP transcription activity was suppressed as HIF-1α transcription factor binds to the promoter of NEP gene (Mitra et al., 2013). Zhang et al. reported a significantly high level of APP, lower level of NEP, increased Aβ accumulation and tau phosphorylation and enhanced activation of astrocytes and microglial cells in the cerebral cortex of prenatal hypoxic AD mice (Zhang et al., 2013). Besides, dysregulation of calcium homeostasis is one of the fundamental mechanisms in AD pathogenesis. Interaction of Aβ with the plasma membrane leads to the formation of calcium-conducting pores, thus elevating cytoplasmic Ca^2+^ concentrations, which enhances neuronal excitation. For example, chronic hypoxia enhances Ca^2+^ entry and mitochondrial Ca^2+^ content by potentiating post-transcriptional trafficking of L-type Ca^2+^ channels (Hassan and Chen, 2021). A previous study reported a significant increase in intracellular Ca^2+^ concentration in the hippocampus areas of mice lacking APP upon hypoxia exposure, and this impairment was attenuated by blocking the L-type Ca^2+^ channels (Hefter et al., 2016).

### Hypoxia and Neuroinflammation

Chronic hypoxia aggravates inflammatory responses in the brain, which in turn promotes the pathogenesis of AD. For instance, previous studies showed that chronic intermittent hypoxia activates microglia to M1 pro-inflammatory phenotype (Zhang et al., 2013; Sapin et al., 2015; Zhang et al., 2017). Moreover, chronic hypoxia was found to induce oxidative stress and production of pro-inflammatory cytokines in the hippocampus and cortex of APPswe/PSEN1dE9 (APP/PS1) mice (Wang et al., 2014) and Sprague Dawley rats (Snyder et al., 2017). In addition to chronic, acute hypoxia was found to increase the production of M1 pro-inflammatory markers, such as the cluster of differentiation 86 (CD86), along with pro-inflammatory cytokines and chemokines, including IL-6, TNF-α, chemokine C-C motif ligand 2 (CCL2) and CCL3 in both hippocampus and cortex of hypoxic APPswe/PS1dE9 transgenic mice compared to normoxic wild-type mice (Zhang et al., 2017). Short-term and acute hypoxia also increased the expression of a subset of pro-inflammatory (TNF-α, IL-1β) and oxidative stress-related (HIF-1α) genes and switched primary rat microglia to M1 pro-inflammatory phenotype (Habib et al., 2014).

Prenatal hypoxia impairs cognitive functions in the postnatal period (Golan and Huleihel, 2006), *via* neurodegeneration, microglial activation and neurotransmitter changes (Bernert et al., 2003). Prenatal hypoxic–ischemic brain injury in P9 mice showed that CD86-positive cells (a marker for M1 pro-inflammatory phenotype) were increased and relative proportion of CD206 (a marker for M2 anti-inflammatory phenotype)-positive cells were reduced after injury, indicating that hypoxia might facilitate M1 polarisation and attenuate M2 activation (Erkenstam et al., 2016). Upon exposure of acute hypoxia, microglial activation in the hippocampus of APPswe/PS1dE9 transgenic mice favoured M1 activation and attenuated M2 activation, concurrently, which resulted in the release of pro-inflammatory cytokines and chemokines, such as IL-6, TNF-α, CCL2 and CCL3, and contributed to the pathogenesis of AD, that is hypoxia-induced neuroinflammation (Zhang et al., 2017). Both hypoxia and Aβ production triggers the activation of microglia, thus leading to a maladaptive neuroinflammatory response, while neuroinflammation itself can initiate the pathogenesis of AD (Lall et al., 2019).

Both hypoxia and neuroinflammation involve multiple signalling pathways cascade including NF-κβ and nuclear factor erythroid 2-related factor 2/haem-oxygenase 1 (Nrf2/HO-1) signalling pathways. These pathways are either activated or inhibited in hypoxia or neuroinflammation.

### NF-κβ Signalling Pathway

NF-κβ is a vital transcription factor that regulates many genes associated with inflammation, innate and adaptive immunity, oxidative stress response and B-cell development (Viatour et al., 2005). NF-κβ stimulates the production of pro-inflammatory cytokines, such as TNFα, IL-1β and IL-6 (Li and Verma, 2002; Yamamoto et al., 2003) and have been shown to be elevated in the brains of AD patients and mice models (Jha et al., 2019). NF-κβ is composed of two subfamilies: ‘NF-κβ’ proteins and ‘Rel’ proteins. NF-κβ is kept in an inactive form in the cytoplasm by the inhibitor of κβ (I-κβ). Various extracellular stimuli (pro-inflammatory cytokines, growth factors, mitogens, microbial components and stress agents) activate NF-κβ, which then forms Iκβ-kinase (IKK) complex. The IKK complex phosphorylates I-κβ proteins that lead to I-κβ degradation mediated by proteasomes. As a consequence, NF-κβ dissociates from I-κβ followed by translocation to the nucleus and initiates the transcription of downstream genes (Viatour et al., 2005; Gilmore, 2006).

Various extracellular stimuli activate the NF-κβ signalling pathway, and hypoxia is one of them. Hypoxia activates the NF-κβ response by tyrosine phosphorylation of I-κβα proteins, which promotes IKK dissociation and NF-κβ DNA binding (Koong et al., 1994). The levels of NF-κβ p50 and p65 subunits, p-Iκβα/Iκβα, as well as nucleoprotein level of NF-κβ p65 increased significantly in transgenic AD mice exposed to hypoxia, associating the increased pro-inflammatory cytokines and chemokines to activation of NF-κβ pathway (Zhang et al., 2017). In another study, resveratrol – a natural phenol with anti-oxidant effects, inhibited the activation of NF-κβ and suppressed the inflammatory reactions in BV2 microglia cells during hypoxia (Song et al., 2014), providing more credence to hypoxia-induced neuroinflammation *via* the NF-κβ-associated signalling pathway. In the context of neuroinflammation, cattle encephalon glycoside and ignotin improved cognitive deficits and suppressed microglia-induced neuroinflammation by increasing brain-derived neurotrophic factor (BDNF) expression and inhibiting TLR4/NF-κβ signalling pathway in the brain of APP/PS1 mice, a mouse model of familial AD (Gao et al., 2021). In a recent study, neuroinflammation and oxidative stress have been reported in neonatal hypoxic brain injuries model using Sprague Dawley rats. The authors found that nuclear NF-κβ (p65) expression was enhanced with substantially lowered cytosolic levels of NF-κβ (p65), indicating active NF-κβ (Yang et al., 2021). Activation of microglial cells and NF-κβ was then significantly downregulated by paeoniflorin, monoterpene glucoside, which is one of the primary bioactive compounds of the *Paeonia lactiflora* plant (Yang et al., 2021).

### Nrf2/HO-1 Signalling Pathway

Nuclear factor erythroid 2-like factor 2 (Nrf2) signalling pathway is one of the defence mechanisms that regulates gene expression of anti-inflammatory mediators and inhibits the progression of neuroinflammation (Buendia et al., 2016; Draheim et al., 2016; Ahmed et al., 2017). Kelch-like ECH-associated protein-1 (Keap1) is a negative regulator which inhibits the transcriptional activity of Nrf2 through ubiquitination and proteasomal degradation under normal physiological conditions (Ahmed et al., 2017). Nrf2 is the principal transcription factor that regulates cell homeostasis related to oxidative and noxious stimuli by mediating the clearance of ROS *via* transcription of phase II anti-oxidant proteins (Wardyn et al., 2015). Various natural products with known anti-inflammatory property successfully reduced the release of pro-inflammatory mediators by improving the Nrf2 level (Alvariño et al., 2018; Luo et al., 2018; Wang et al., 2018; Xu et al., 2019; Zhou et al., 2019; Ma et al., 2020). Moreover, knockdown Nrf2 using Nrf2 siRNA in BV2 microglial cells has significantly reversed the inhibitory effects of cryptotanshinone (CTN), a monomer compound extracted from the dried roots and rhizomes of Salvia miltiorrhiza, on the release of M1 pro-inflammatory mediators, such as NO, cyclooxygenase 2 (COX2), inducible nitric oxide synthase (iNOS), IL-1β, IL-6 and TNF-α. These findings indicate that activation of Nrf2 directly regulates neuroinflammation (Zhou et al., 2019).

HO-1 is a target gene of Nrf2 that responsible for the rate-limiting step in the degradation of free haem into carbon monoxide (CO) and free iron and biliverdin to bilirubin by biliverdin reductase (BVR). The production of anti-inflammatory mediators, such as CO and bilirubin, and degrading pro-inflammatory mediators, such as free haem, play important roles in providing the protective effects of HO-1 towards inflammation (Yang et al., 2013; Ahmed et al., 2017). One study reported that both HO-1 and BVR were increased in the plasma of probable AD patients, and HO-1 is a systemic marker in early sporadic AD (Nitti et al., 2018). Many studies have demonstrated that HO-1 and its metabolites exhibited significant anti-inflammatory and neuroprotective effects, which are mediated by Nrf2 (Li et al., 2014). Increase in HO-1 expression in dendritic cells inhibited the LPS-induced release of pro-inflammatory cytokines (Chauveau et al., 2005). Meanwhile, in a male Sprague Dawley rat liver transplantation model, activation of Nrf2 caused the elevation of HO-1 expression, which then inhibited the NF-κβ signalling pathway (Chi et al., 2015).

Administration of quercetin in combination with sitagliptin significantly improved cognitive impairments caused by Aβ infusion in rats. The effect was accompanied by a reduction in Aβ1-42 level, decreased in malondialdehyde level, increased in anti-oxidant enzymatic activity and increased expression of Nrf2/HO-1 signalling pathway in the rat brain, suggesting its neuroprotective effect in AD (Li et al., 2019). A previous study used isorhamnetin, the most abundant flavanol in sea buckthorn (*Hippophae rhamnoides L*.) showed its significant cytoprotective effects against H_2_O_2_-induced mouse-derived C2C12 myoblast cells by upregulating Nrf2-mediated HO-1 expression and scavenging ROS (Choi, 2016). The upregulation of Nrf2-mediated HO-1 expression also prevented acute inflammation, which induced by LPS, in mouse peritoneal macrophage-derived foam cell (Kuhn et al., 2011) as well as increased the efferocytic activity of murine macrophages treated with taurine chloramines (Kim et al., 2015). The Nrf2/HO-1 pathway also plays an important role in neuroinflammation induced by LPS in mouse BV2 microglial cells and mouse hippocampal HT22 cells. For instance, upregulation of HO-1 expression through the Nrf2 pathway in BV2 microglial cells was able to protect hippocampal HT22 from neuroinflammation-mediated toxicity that caused the cells death (Lee and Jeong, 2014).

## Hypoxia-Induced Neuroinflammation: Human Alzheimer’s Disease Clinicopathological Evidence

Normal ageing causes brain atrophy and deterioration of brain circulatory functions, including a reduction in CBF and an impairment in oxygen metabolism (Zlokovic, 2011). Indeed, ageing is a significant risk factor for the development of most neurodegenerative diseases, including AD (Hou et al., 2019). According to a recent study, the metabolic vulnerability of healthy human brain to hypoxia increases with age, as evidenced by decreased cerebrovascular reactivity (CVR) and cerebral metabolic rate of oxygen (CMRO_2_) and increased lactate concentration, as measured by functional magnetic resonance imaging (MRI) and spectroscopy (Vestergaard et al., 2020). Similarly, studies in AD patients using arterial spin-labelling MRI and transcranial Doppler ultrasonography revealed a loss in CBF and impaired CVR as well as altered cerebral metabolic parameters in their brains (Schuff et al., 2009; De Heus et al., 2018), that are associated with decreased oxygen consumption and therefore, indicating a hypoxic environment in the brain. Cerebral hypoxia is a condition that can happen either as global hypoxia or as cerebrovascular accident or ischemic stroke, due to a broad set of clinical pathologies, such as myocardial infarction, cerebral small vessel disease (CSVD), arteriosclerosis, diabetes mellitus or even genetic disorders (Merelli et al., 2020; Bandyopadhyay, 2021). Moreover, some other conditions that lead to a reduced supply of O_2_ in the brain include anaemic hypoxia, pulmonary ventilation insufficiency or obstruction defects, obstructive sleep apnoea, neonatal asphyxia, hypobaric altitude hypoxia and carbon monoxide inhalation (Merelli et al., 2020). All of these pathologies can be aggravated by additional factors (for instance, poor lifestyle habits and smoking), leading to neuronal death and eventually cognitive decline (Merelli et al., 2020).

Since AD is clinically manifested by memory loss and cognitive decline, these hypoxic insults are considered as predisposing factors of ageing brain towards AD (Zhang et al., 2019). A spatial and episodic memory of AD patients is progressively deteriorated over time as the brain processes in their hippocampus become dysfunctional and very sensitive to hypoxia (Shaw et al., 2021). The changes in vascular structures and functions are commonly observed as early features of developing AD, as the dysfunction of blood–brain barrier and inadequate cerebral perfusion can promote accumulation of Aβ as well as hyperphosphorylation of tau proteins (Montagne et al., 2016). The mild cognitive impairment patients without AD pathology had a lower local blood volume in their hippocampus, indicating a reduced hippocampal perfusion is associated with cognitive decline (Wang et al., 2006). Therefore, adequate oxygen delivery in the hippocampus is vital for efficient neuronal function and signalling, thus preventing cognitive impairment.

Apart from their association with ageing, cerebrovascular diseases and AD share a series of risk factors and neuropathological similarities (Love and Miners, 2016). Studies in patients resuscitated after cardiac arrest (transient cerebral hypoperfusion) revealed an elevation of Aβ levels in their blood (Zetterberg et al., 2011; Pluta et al., 2021) and an overexpression of APP in their cortical and subcortical neurons (Wiśniewski and Maślińska, 1996), emphasising the significant of hypoxia in the amyloidogenic process of AD. Furthermore, a systematic review of CSVD and AD-related studies highlighted some specific CSVD neuroimaging markers, such as white matter hyperintensities and microinfarct are associated with an increased risk of AD. However, these results are still inconclusive because further investigation is needed to understand the relationship between these two diseases (Liu et al., 2018). There is increasing evidence that sleep-disordered breathing (SDB), which is associated with intermittent hypoxia and sleep fragmentation can increase the risk of developing AD syndrome. A cross-sectional study conducted between 2016 and 2018 in France using data from the Age-Well randomised clinical trial shown that cognitive-unimpaired older individuals with untreated SDB had greater brain changes in terms of amyloid deposition, grey matter perfusion, volume and metabolism, which overlapped over AD-sensitive brain regions, including posterior cingulate, cuneus and precuneus areas (André et al., 2020). Hence, a risk for AD can be reduced by early screening and treatment of SDB, particularly in asymptomatic elderly.

In addition, there is a hypothesis called two-hit vascular hypothesis of AD, in which the existence of first hit, vascular contribution followed by second hit, Aβ accumulation (Zlokovic, 2011). Based on this hypothesis, vascular risk factors, like hypertension or diabetes, may lead to neurovascular unit (brain endothelial cells, pericytes, vascular smooth muscle cells, glial cells and neurons) dysregulation and hypoxia (Soto-Rojas et al., 2021). An imbalance between Aβ production and clearance is worsened in hypoxic state, leading to vascular and parenchymal Aβ accumulation. These two hits interact dynamically, triggering a cascade of events that precedes dementia, which includes neurofibrillary tangle formation, neuronal dysfunction and accelerates neurodegeneration, recognised as AD pathologies (Soto-Rojas et al., 2021). Neuroinflammation has been linked to brain damage caused by hypoxic insults (Chen et al., 2009) and is implicated in the pathogenesis of AD. Post-mortem analysis revealed a consistent increase in expression of microglial activation markers, major histocompatibility complex II (MHCII) and cluster of differentiation 68 (CD68) in the brains of AD patients, when compared to other markers that are commonly expressed in both resting and activated microglia, such as ionised calcium-binding adaptor molecule 1 (Iba-1) and cluster of differentiation 11b (CD11b), which were not elevated (Hopperton et al., 2018). In line with this, a histological finding from a post-mortem of 22-year-old with severe hypoxic–ischemic brain damage found that majority of activated microglia with CD68 markers surrounded the damaged neurons in the hippocampus (Rahaman and Del Bigio, 2018). Other than that, human leukocytes antigen (HLA-DR) marker also was shown on activated microglia which phagocytosed the damaged neurons in the cerebellum of 60-year-old with severe hypoxic brain injury (Rahaman and Del Bigio, 2018).

Neuroimaging is essential for diagnosis of vascular cognitive impairment and dementia, an AD-like pathology, because it can assess the type of lesion, location and severity of the condition in the brain. The development of neuroimaging techniques discovered multiple regions in the brain that are prone to AD pathology showed a significant deterioration in CBF even at the stage of mild cognitive impairment (MCI), which involves multifactorial processes leading to demyelination and gliosis (Salminen et al., 2017; Frantellizzi et al., 2020). The multifactorial processes are including blood–brain barrier disruption, hypoxia and hypoperfusion, oxidative stress, neuroinflammation and alteration on neurovascular unit coupling, cerebral microbleeds or superficial siderosis (Frantellizzi et al., 2020). The MRI and positron emission tomography (PET) imaging are the most used and reliable techniques to localise the lesion in the brains of AD patients. However, a combination of both provides a better result in diagnosing the cognitive disorders, particularly AD, with MRI showing white matter lesions, while PET demonstrating cerebral glucose metabolism impairment and deposition of Aβ (Frantellizzi et al., 2020). Based on these techniques application and histopathological observations, an extensive cerebral amyloid angiopathy (CAA) was observed in the hippocampus and other regions of cerebral cortex of AD patients’ (Salminen et al., 2017). CAA is characterised by a decrease in CVR due to a vasodilatory response, and it affects 82-98% of patients with AD (Switzer et al., 2020). Furthermore, the first investigation on CSVD and *in vivo* neuroinflammation using [^11^C]PK11195 PET imaging revealed an association between microglial activation and CSVD in MCI and early-stage AD patients (Low et al., 2020). In contrast, the collection of cerebrospinal fluid is an invasive procedure that offers a valuable information on Aβ and tau proteins concentration in the brains of AD patients (Ausó et al., 2020). The presence of these specific biomarkers of AD and other biomarkers of AD-related (synaptic dysfunction, neuroinflammation, oxidative stress and more) in CSF is being extensively studied since they can predict the cognitive symptoms before it develops (Ausó et al., 2020; Ferreira et al., 2014). Interesting result was obtained by Kester and co-workers (2014) in cross-sectional studies examining the relationship of MRI white matter hyperintensities, lacunes and microbleeds with levels of CSF Aβ42, total tau and tau phosphorylated at threonine 181 (P-tau_181_) in patients with AD, patients with vascular dementia and control participants. Results from this clinical study indicate that deposition of amyloid was aggravated in CSVD patients with apolipoprotein E carriers (Kester et al., 2014).

### Neuroprotective Effects of *Centella asiatica*

At present, AD is the fourth biggest cause of death after cardiovascular disease, cancer and stroke. The current AD drugs offer transient symptomatic improvement but have little effect on modifying the long-term pathophysiology of this disease. Given the lack of success in these synthetic drugs research, efforts are being directed towards the development of alternative treatment strategies, including the use of natural products as anti-AD therapy. Many systematic reviews revealed the natural products and/or their natural active compounds have great neuroprotective potential in the prevention and/or treatment of AD (Auti and Kulkarni, 2017; D’Onofrio et al., 2017; Andrade et al., 2019; Noori et al., 2021). Although the scientific evidence from the preclinical studies of AD is promising, but the clinical trials are still required to validate their safety and efficacy in humans. Nevertheless, the natural products have gained popularity in recent years because they are less toxic, have fewer side effects and are inexpensive than the synthetic drugs.

*Centella asiatica* (CA) and its key phytochemical composition have been reported to exert multiple health-beneficial effects including anti-oxidant, anti-inflammation, anti-bacterial, wound healing and enhancement of cognition and memory (Hashim, 2011; Seevaratnam et al., 2012). Traditionally, CA is eaten raw or cooked and taken as juice, tonic drink or herbal tea to treat many kinds of diseases, such as gastrointestinal problems, gastric ulcer, asthma and eczema (Brinkhaus et al., 2000). CA contains alkaloids, carbohydrates, amino acids, fatty acids, minerals, vitamins, terpenes, flavonoids and phenolic compounds (Gohil et al., 2011; Gray et al., 2018; Ogunka-Nnoka et al., 2020). The major group of the phytochemical composition in CA is pentacyclic triterpenoids, namely, madecassoside, asiaticoside, madecassic acid and asiatic acid (Gray et al., 2018; Razali et al., 2019; Figure 2). These triterpenes contribute to the pharmacological activities of this medicinal plant (Gohil et al., 2011; Sun et al., 2020). The ethanolic extract of CA ancessions has been reported to contain a significant amount of madecassoside and asiaticoside with only a few madecassic acid and asiatic acid was retained in the extract (Table 1; Jusril et al., 2020). Similarly, other standardised extract of CA, named ECa 233, contains at least 80% of those triterpenoids, in which madecassoside and asiaticoside appeared to be 51 and 35% in the extract (Hengjumrut et al., 2017). Both madecassoside and asiaticoside are known to be the parent compound of their aglycones, madecassic acid and asiatic acid, respectively (Songvut et al., 2019). However, these parent compounds is bio-transformed into their hydrophobic active metabolites before being absorbed in the intestine and become predominant compounds found in the plasma and tissues (Songvut et al., 2019). The clinical pharmacokinetics of these triterpenoids have been discussed elsewhere in detail (Sun et al., 2020; Songvut et al., 2021).

**Figure 1:**
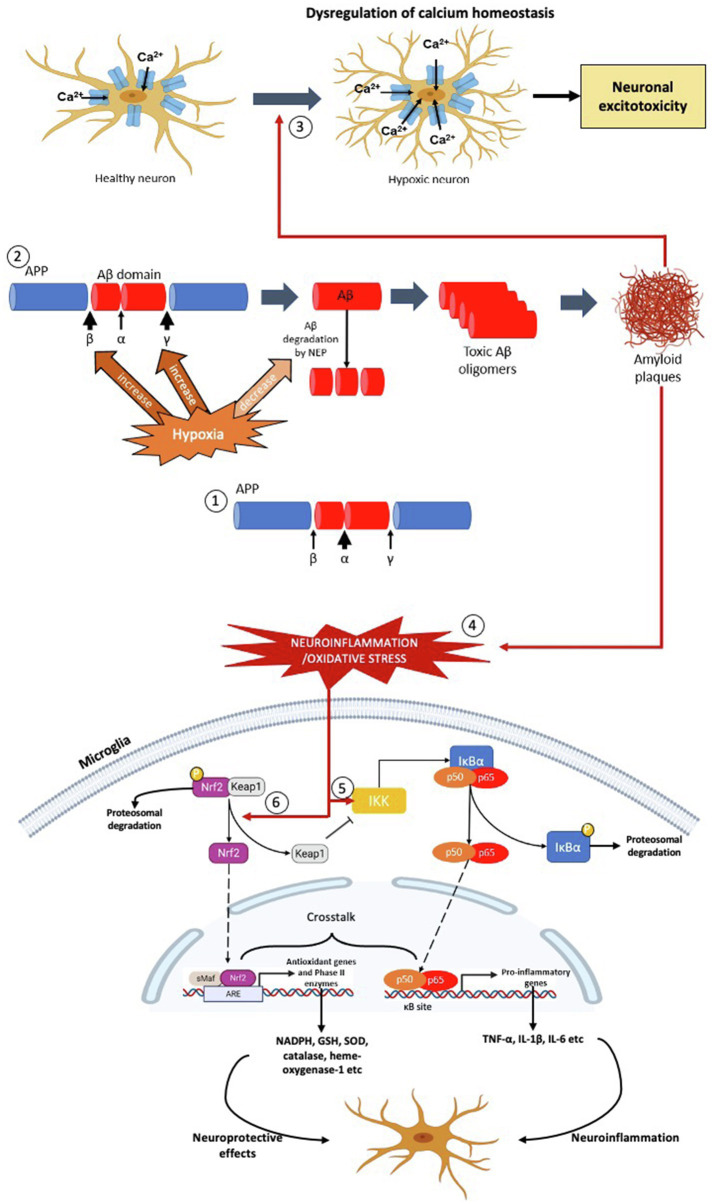
The effects of hypoxia on formation of Aβ plaques, oxidative stress and neuroinflammation – the pathophysiology of AD. (1) In normoxia, the formation of Aβ peptide is less due to the α-secretase cleaves at the middle of the Aβ domain. (2) In hypoxic condition, the activities of β- and γ-secretase are increased resulting in production of Aβ peptide. Concurrently, hypoxia also decreases the activity of neprilysin (NEP) in degrading the Aβ peptide. As a result, more toxic Aβ oligomers are produced which leads to formation of Aβ plaques. (3) The Aβ plaques dysregulate the calcium homeostasis of the neurons which increase the calcium ions influx, leading to mitochondrial dysfunction, protease and lipase activation and osmotic swelling, eventually cause neuronal excitoxicity. (4) The formation of Aβ plaques causes neuroinflammation and oxidative stress, leading to activation the NF-κβ and Nrf2 signalling pathways in the microglial cell. (5) Neuroinflammation activates Iκβ-kinase (IKK), which leads to phosphorylation of inhibitor of NF-κβ, I-κβ and hence targets the later for polyubiquitination-mediated proteasomal degradation. As a result, the NF-κβ dimer (p50 and p65) is released, which then translocates into the nucleus, binds with the NF-κβ response elements of the genome. This leads to transcription of pro-inflammatory genes, such as TNF-α, IL-1β and IL-6 which aggravate the neuroinflammatory response and oxidative stress. (6) On the other hand, oxidative stress activates Nrf2 signalling pathway by dissociating Nrf2 from its inhibitor, Keap1. In normal condition, Nrf2 is bound to cytosolic repressor Kelch-like ECH-associated protein 1 (Keap1) and labelled for polyubiquitination-mediated proteasomal degradation. However, during neuroinflammation and/or oxidative stress, the Nrf2 is released from Keap1, which then translocates into the nucleus, binds with anti-oxidant response elements of the genome along with small Maf proteins. This leads to transcription of anti-oxidant genes and phase II enzymes, such as NADPH, GSH, SOD, CAT and HO-1, which exhibit neuroprotective effects on neuronal cells.

**Table 1:** The total concentration of pentacyclic triterpenoids and their AChE inhibitory activities in the SECA (Jusril et al., 2020).

Triterpenes of CA	Concentration of triterpenes (mg/g)	AChE Inhibitory activity, IC_50_ Values (μg/ml)
Asiaticoside	105.71	59.13±0.18
Madecassoside	179.64	37.14±0.04
Asiatic acid	132.26	15.05±0.05
Madecassic acid	112.82	17.83±0.06

**Figure 2:**
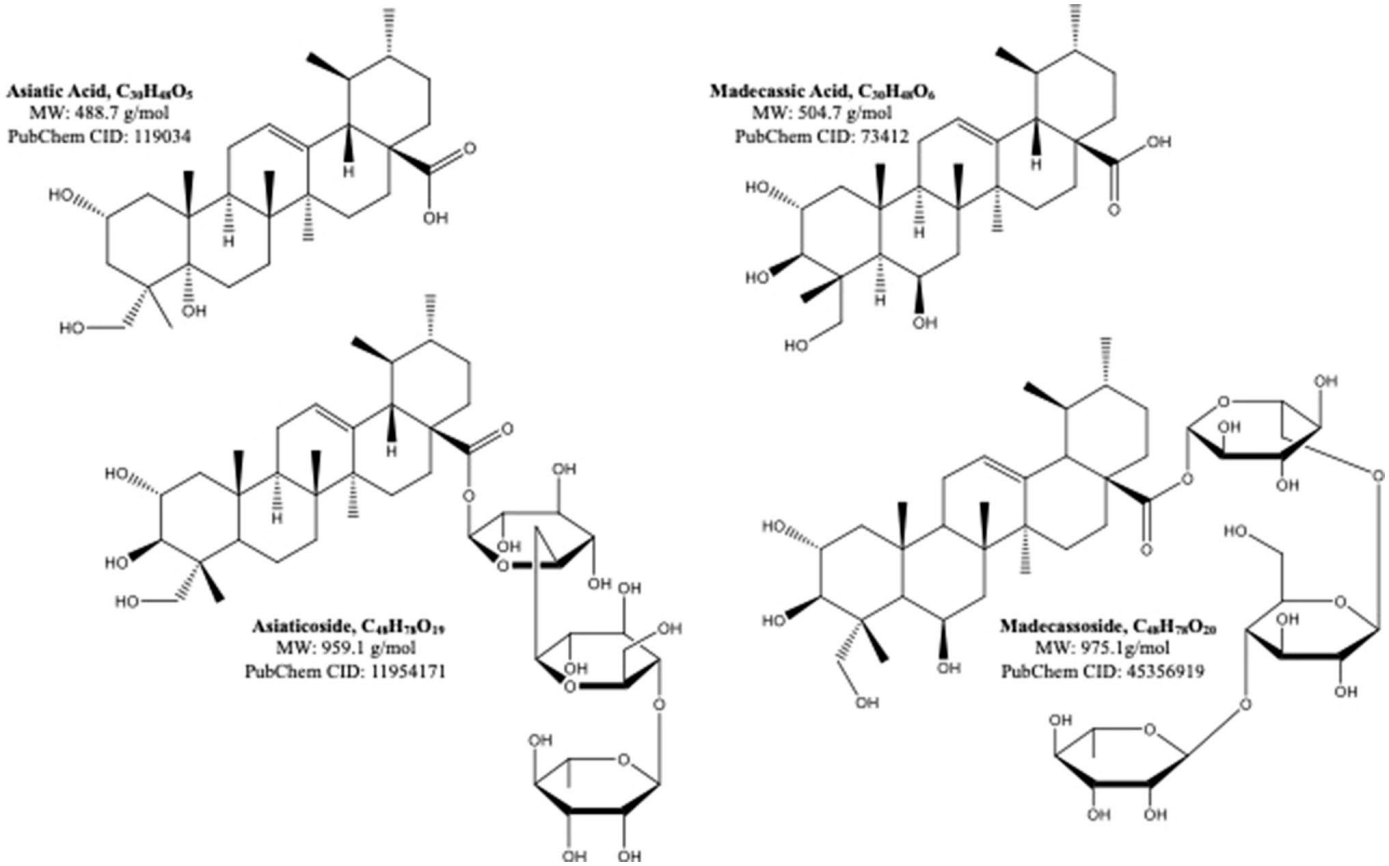
The molecular structures of pentacyclic triterpenoids of *Centella asiatica* (Sun et al., 2020).

**Figure 3:**
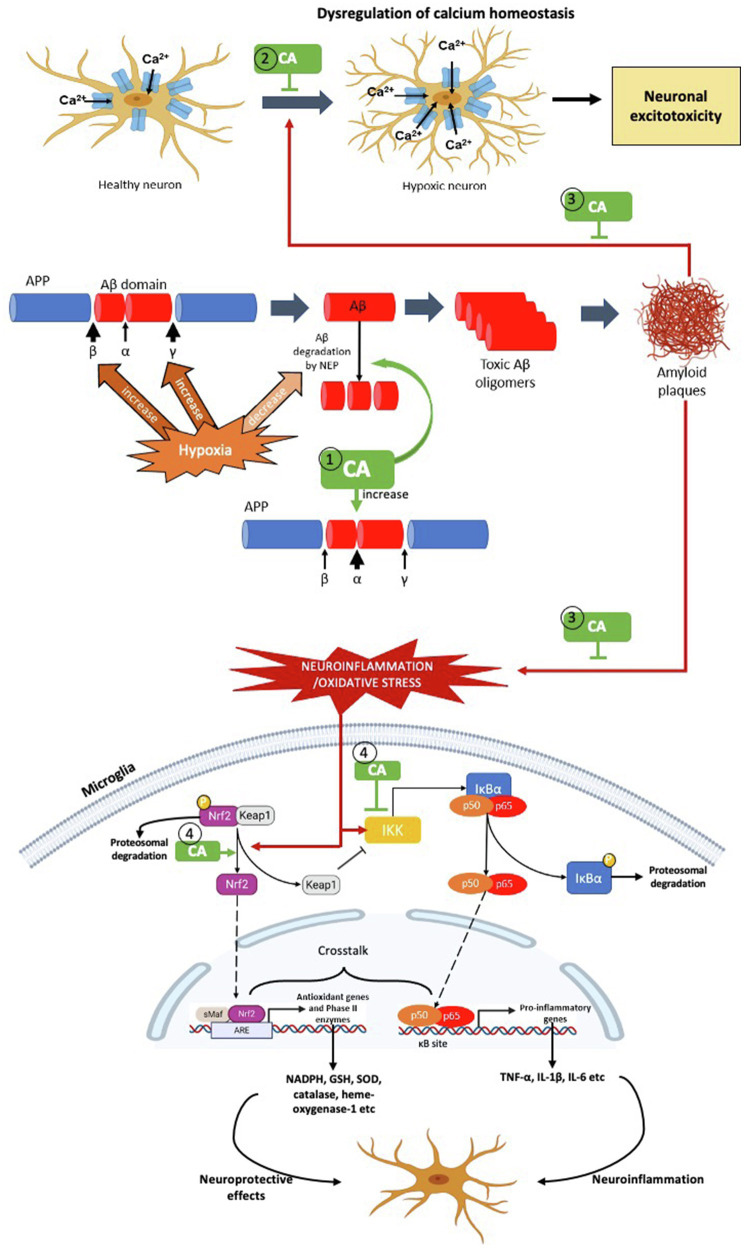
The possible targets of *Centella asiatica* as neuroprotection in hypoxia-induced neuroinflammation – the pathophysiology of AD. We propose several targets for *Centella asiatica* (CA) which (1) suppresses the formation of Aβ plaques (2) inhibits or suppresses the dysregulation of calcium homeostasis (3) attenuates the effect of Aβ plaques formation towards the dysregulation of calcium homeostasis, oxidative stress and neuroinflammation and (4) attenuates the activation of NF-κβ signalling pathway as well as enhances the activation of Nrf2 signalling pathway, resulting in neuroprotection.

As aforementioned, the inhibitory effect against AChE enzyme plays a pivotal role in restoring the cholinergic functions in AD patients, thus improving their cognitive and memory functions (Talesa, 2001; Briggs et al., 2016). Several preparations of CA extracts including butanol, ethyl acetate and hexane were found to normalise the level of acetylcholine as well as AChE activities in the CNS of seizure-induced rats’ model (Visweswari et al., 2010). More recently, a raw extract of CA (RECA) significantly inhibited the AChE activity in human neuroblastoma cells. However, the anti-AChE activity was insignificantly reduced in the *in vivo* model of LPS-induced neuroinflammation. Interestingly, this study demonstrated that the level of pro-inflammatory cytokines and oxidative stress was significantly decreased by RECA in a concentration-dependent manner in LPS-stimulated murine microglial cells and LPS-induced rats (Hafiz et al., 2018). Furthermore, this research team reported that asiatic acid and madecassic acid exerted a more efficient AChE inhibitory activity by using Ellman’s spectrophotometer method (Table 1; Jusril et al., 2020), but yet to be confirmed in *in vitro* and *in vivo* models. Theoretically, these pentacyclic triterpenic acids were also demonstrated to possess strong hydrogen and hydrophobic bonds in the ligand-enzyme interactions, as evaluated in the molecular docking analysis (Jusril et al., 2020).

The anti-oxidant properties of CA contributed to its neuroprotective effects against Aβ_1-40_-induced neuronal damage in rat PC12 pheochromocytoma and human IMR32 neuroblastoma cells (Chen et al., 2016). It is noteworthy that the neuroprotective effect was mediated by activation of anti-oxidative defence systems and subsequent reduction in ROS formation (Chen et al., 2016). In another preclinical *in vitro* studies, two triterpenes derived from CA – asiatic acid (Qian et al., 2018) and madecassoside (Sasmita et al., 2018), reduced neuroinflammation in LPS-induced microglial cells by reducing NF-κβ p65 acetylation and thereby preventing NF-κβ activation (Qian et al., 2018) in addition to increased expression of anti-neuroinflammatory HO-1 expression (Sasmita et al., 2018). Similarly, recent *in vitro* analyses conducted by Mairuae et al. (2019) showed that CA extract decreased production of NO, TNF-α and ROS in LPS-stimulated microglial cells. The anti-inflammatory effects were related to the suppression of NF-κβ p65 translocation and the inactivation of the PI3K/AKT and ERK1/2 signalling pathways (Mairuae et al., 2019). Furthermore, the neuroprotective effect of asiaticoside was reported in human brain microvascular endothelial cells (hBMECs), the main component of blood–brain barrier (BBB) that is connected with tight junctions, and both components play an important role in maintaining the BBB integrity (Abbott et al., 2010). The disruption of BBB integrity is believed to be associated with the pathogenesis of AD by abnormally exchanging the Aβ from blood to CNS, causing abnormal deposition of Aβ in the brain (Bachmeier et al., 2010). A study by Song et al. (2018) found that asiaticoside significantly attenuated apoptosis and cytotoxicity and improved mitochondrial membrane potential in Aβ_1-42_-induced hBMECs. Mechanistically, the authors reported that asiaticoside significantly inhibited translocation of NF-κB p65 from cytoplasm to the nucleus and downregulated the expression of TNF-α, IL-6, TLR4, MyD88, TRAF6 and p-NF-κB p65 in a concentration-dependant manner in hBMECs (Song et al., 2018). In general, these studies support the notion that the neuroprotective effects of CA, either as a whole extract or its major phytochemicals, could be mediated by the TLR4/NF-κB, PI3K/AKT and ERK1/2 signalling pathways.

Preclinical *in vivo* studies have further supported the neuroprotective effect of *CA*. For instance, Chiroma et al. (2019) demonstrated that CA extract improved cognitive abilities in D-galactose/aluminium chloride (AlCl_3_)-induced cognitive deficits of male albino Wistar rats. Additionally, the CA extract restored cholinergic dysfunction by reducing AChE level, attenuated oxidative stress by lowering malondialdehyde (MDA) and increasing superoxide dismutase (SOD) levels in the hippocampus and ameliorated cognitive impairment by inhibiting aberrations of ultrastructural morphology of neurons in the prefrontal cortex (Chiroma et al., 2019). Likewise, the asiatic acid treatment significantly attenuated aluminium overloading, AChE hyperactivity, behavioural impairment, Aβ burden and inflammation in AlCl_3_-induced male albino Wistar rats (Rather et al., 2018). In addition, a study done by Gray’s team demonstrated that CA water extract (CAW) successfully improved Morris water maze performance in aged C57BL/6 mice group, in comparison with the young C57BL/6 mice group. Interestingly, the CAW significantly increased the expression of mitochondrial and anti-oxidant response genes in the hippocampus, frontal cortex and cerebellum of these mice regardless of their ages (Gray et al., 2016).

While numerous preclinical *in vitro* and *in vivo* experiments have been undertaken to access the neuroprotective effects of CA against AD, only a few clinical studies have been reported (reviewed in Lokanathan et al., 2016; Gray et al., 2018; Sun et al., 2020; Wong et al., 2021). In one clinical study, Tiwari et al. (2008) prescribed powdered CA extract to elderly with mild cognitive impairment (MCI) for six months. The authors discovered that CA significantly improved the mean scoring of Mini Mental State Examination (MMSE) after six months prescription of CA, together with improved diastolic blood pressure, peripheral neuritis, insomnia and loss of appetite among the MCI elders (Tiwari et al., 2008). A recent systematic review and meta-analysis of 11 randomised controlled trials demonstrated there was no significant relationship between the use of CA and cognitive function improvement (Puttarak et al., 2017). However, several methodological limitations, such as dose regimen, plant preparation, standardisation and product variation, have been identified, which may have an impact on the quality of findings (Puttarak et al., 2017). Therefore, the future clinical trials need to be thoroughly prepared to achieve best results on the effect of CA on cognitive functions in humans.

Rather than having its remarkable neuroprotective effects, CA and/or its phytochemical composition also exhibits some other protective effects on periphery which results in lowering the risk of AD development. For instance, hypertension is one of the risk factors that can accelerate the late-onset AD. Previous study done by Thiwarapan et al. (2019) shown that the juice of CA leaves significantly reduced the blood pressure and heart rate as well as improved the regional cerebral blood flow in both normal and hypertensive rat groups (Thirawarapan et al., 2019). Similarly, a study on anti-hypertensive effect of CA has been conducted in hypertension patients which showed that their systolic and diastolic blood pressure were significantly reduced after consuming the CA herbal tea (Astutik et al., 2021). Moreover, the asiatic acid has been reported to improve blood pressure in a hypertensive *in vivo* model by decreasing renin angiotensin overactivity, sympathetic nerve overactivity and improving vascular function and NO bioavailability (Maneesai et al., 2016, 2017). Similarly, the asiaticoside exhibit anti-hypertensive effect by inhibiting a raised blood pressure and improving NO production and cGMP in hypoxia-induced pulmonary hypertensive rat models (Wang et al., 2015, 2018). Unfortunately, clinical research on the anti-hypertensive effects of madecassoside and madecassic acid is still lacking. Both preclinical and clinical studies on neuroprotective effects of CA as mentioned above provide an understanding that this medicinal plant as an extract and/or its major phytochemical composition can be a potential therapeutic agent of neurodegenerative diseases, particularly AD.

Flurbiprofen (FP), a non-steroidal anti-inflammatory drug, is one of FDA-approved drugs in the market that has been repurposed to treat AD due to its activity in modulating γ-secretase (Crump et al., 2013; Al-azzawi et al., 2020). Specifically, FP can selectively modulate the γ-secretase activity, without compromising the other APP processing pathways, resulting in the reduction of Aβ42 levels (Meister et al., 2013). Despite being a well-absorbed and short half-life drug (Davies, 1995), FP has a low penetration across BBB and unable to achieve a required concentration to elicit its pharmacological effect on γ-secretase activity in the brain (Wilcock et al., 2008; Green et al., 2009). Previous studies used nanocarriers in transporting the FP across BBB in order to improve the drug liberation (Crump et al., 2013; Al-azzawi et al., 2020), yet the bioavailability of the drug is still insufficient to produce its pharmacological impact in the targeted brain areas. In the context of CA, a recent study using *in vitro* BBB model from primary porcine brain endothelial cells demonstrated that its phytochemicals, in particular to asiaticoside, madecassoside and asiatic acid, are efficacious in crossing the BBB without producing any toxic effects and obstructing the integrity of BBB tight junction (Hanapi et al., 2021). Hence, the high bioavailability of CA and its key phytochemical composition in the brain provides more insight into the development of a potential neurotherapeutic agent for AD in the future.

The neuroprotective effects of CA have not been so extensively studied in hypoxia-induced model. To date, asiaticoside, one of the triterpenes derived from CA, has been reported to exert its neuroprotective effects towards the *in vitro* model of cerebral ischemia by exposing primary cultured newborn rat cortical neurons to hypoxia. The authors found that asiaticoside significantly increased the cell survival rate, reduced lactate dehydrogenase release and inhibited neuronal cell apoptosis by modulating the expression of apoptotic factors, including Bcl-2, Bax and caspase-3 in hypoxia-induced primary cultured newborn rat cortical neurons (Sun et al., 2015). Meanwhile, in a mouse model of permanent cerebral ischemia, asiatic acid of CA significantly reduced infarct volume and improved neurological deficit scores, possibly through its anti-oxidant and anti-inflammatory effects. In the same study, the authors showed that asiatic acid significantly improved cell survival and enhanced mitochondrial membrane potential in an oxygen–glucose deprivation-induced HT-22 hippocampal neuronal cell line (Krishnamurthy et al., 2009). Similarly, a combination of CA extracts and *Acalypha indica L* (AI) decreased the neuronal cell damage in the hippocampus of hypoxia-induced injury rats model (Farida et al., 2018). Furthermore, a later finding showed that CA extract exhibited its anti-oxidant effects by attenuating the reduction of body length in the hypoxia-exposed zebrafish group (Ariani et al., 2019). Notably, the hypoxic condition causes an increase in oxidative stress and eventually harms the mitochondria in the brain, which leads to growth retardation as body metabolism processes decrease in zebrafish. Based on the above-mentioned studies, it appears that CA and/or its phytoconstituents have potential neuroprotective effects in hypoxia-induced models; however, yet to date, there is still a gap in the knowledge on the neuroprotective effects of CA against hypoxia-induced neuroinflammation. The studies presented thus far provide evidence that the correlation between hypoxia and neuroinflammation in AD model can be a useful tool to outline the neuroprotective effects of CA against hypoxia-induced neuroinflammation.

## Conclusion and Future Directions

In general, given its complex pathogenesis, targeting therapy for AD would likely to implicate a multi-model approach due to the variety of mechanisms and pathways involved. Previous studies reported that neuroinflammation is one of AD hallmark pathophysiological features and is increasingly recognised as a potential therapeutic target of this neurodegenerative disease, recently. It has been shown that neuroinflammation can be acquired in the hypoxic condition *via* several pathways, including NF-κβ and Nrf2. These two pathways had been reported to play important role in regulating neuroinflammation *via* suppression of NF-κβ pathway and activation of Nrf2 pathway which attenuating pro-inflammatory mediators, such as TNF-α, IFN-γ, IL-1β and IL-6 as well as enhancing anti-oxidant proteins, such as SOD, CAT, HO-1, GSH and NADPH. It is noteworthy to mention that hypoxia is not only able to disturb metabolic balance of Aβ, but also aggravates Aβ toxicity by causing calcium dyshomeostasis and elevating ROS, that eventually leading to neuronal cell death and ultimately, dementia.

*Centella asiatica* (CA) is a medicinal plant that contains multiple active phytochemicals – majority in pentacyclic triterpenoids group include asiatic acid, asiaticoside, madecassic acid and madecassoside. Among these triterpenes, asiatic acid is the most extensively studied in hypoxia model, followed by asiaticoside and madecassic acid. In contrast, madecassoside is the least studied in hypoxia model, despite it being reported to be abundantly found in CA extract. Therefore, further research should consider the pharmacological activity of madecassoside in the hypoxia model. Although the neuroprotective properties of CA have been well-studied and documented, the impact of CA extract and/or its specific phytochemicals in hypoxia-induced neuroinflammation should be explored in detail. In this review, we emphasise several potential therapeutic targets for *Centella asiatica* to confer neuroprotection by suppressing the formation of Aβ plaques, attenuating the activation of NF-κβ signalling pathway and enhancing the activation of Nrf2 signalling pathway (Figure 3). Not limited to these two pathways, several other possible molecular mechanisms and/in different signalling pathways, including upstream and downstream regulation of PI3K/AKT and apoptotic pathways need to be considered that associated with the effects of CA and/or its phytochemical composition. Other gaps include the need to investigate and validate those molecular mechanisms and signalling pathways before we can make headways for clinical trials regarding the effectiveness of *CA*. Strikingly, the phytochemicals of CA have high permeability across blood–brain barrier – a significant obstacle in developing new neurotherapeutic agent. This discovery provides a better future of *Centella asiatica* as an agent in therapeutic strategies or interventions to target multiple mechanisms and/or pathways against the neurodegenerative disease, particularly AD.

## Author Contributions

AH drafted, prepared the figures and table, and revised the manuscript. HH conceived the original idea, drafted, reviewed, and critically revised the manuscript. JK, NH, SM, MZM, MC, MM, MA, and JS critically revised the manuscript for important intellectual content. All authors contributed to the article and approved the submitted version.

## Funding

The study was funded by Universiti Putra Malaysia – Putra Grant (GP/2017/9566200).

## Conflict of Interest

The authors declare that the research was conducted in the absence of any commercial or financial relationships that could be construed as a potential conflict of interest.

## Publisher’s Note

All claims expressed in this article are solely those of the authors and do not necessarily represent those of their affiliated organizations, or those of the publisher, the editors and the reviewers. Any product that may be evaluated in this article, or claim that may be made by its manufacturer, is not guaranteed or endorsed by the publisher.
